# Clinical features of anti-synthetase syndrome associated interstitial lung disease: a retrospective cohort in China

**DOI:** 10.1186/s12890-021-01399-5

**Published:** 2021-02-12

**Authors:** Xi Zhan, Wei Yan, Ying Wang, Qing Li, Xuhua Shi, Yanli Gao, Qiao Ye

**Affiliations:** 1 Beijing Institute of Respiratory Medicine, Department of Respiratory Medicine and Critical Care, Beijing Chaoyang Hospital, Capital Medical University, Beijing, 100020 China; 2 Hunan Prevention and Treatment Institute for Occupational Diseases, Changsha, 410007 Hunan China; 3 Department of Pathology, Beijing Chaoyang Hospital, Capital Medical University, Beijing, 100020 China; 4 Department of Rheumatology and Autoimmune Diseases, Beijing Chaoyang Hospital, Capital Medical University, Beijing, 100020 China; 5 Department of Radiology, Beijing Chaoyang Hospital, Capital Medical University, Beijing, 100020 China; 6 Department of Occupational Medicine and Toxicology, Capital Medical University, Beijing, 100020 China

**Keywords:** Anti-synthetase syndrome, Interstitial lung disease, Organizing pneumonia, Nonspecific interstitial pneumonia

## Abstract

**Background:**

Anti-synthetase syndrome (ASSD) is a chronic autoimmune condition characterized by antibodies directed against an aminoacycl transfer RNA synthetase (ARS) along with a group of clinical features including the classical clinical triad: inflammatory myopathy, arthritis, and interstitial lung disease (ILD). ASSD is highly heterogenous due to different organ involvement, and ILD is the main cause of mortality and function loss, which presents as different patterns when diagnosed. We designed this retrospective cohort to describe the clinical features and disease behaviour of ASSD associated ILD.

**Methods:**

Data of 108 cases of ASSD associated ILD were retrospectively collected in Beijing Chaoyang Hospital from December 2017 to March 2019. Data were obtained from the Electronic Medical Record system. Patients were divided into 5 groups according to distinct aminoacyl tRNA synthetase (ARS) antibodies.

**Results:**

Overall, 108 consecutive patients were recruited. 33 were JO-1 positive, 30 were PL-7 positive, 23 were EJ positive, 13 were PL-12 positive and 9 were OJ positive. The JO-1 (+) group had a significant higher rate of mechanic’s hand (57.6%) than other 4 groups. Polymyositis/dermatomyositis (PM/DM) was diagnosed in 25 (23.1%) patients and no difference was observed among the 5 groups. The PL-7 (+) group had a higher frequency of UIP pattern (13.3%) than the other 4 groups but the difference was not significant, and the EJ (+) group had the most frequent OP pattern (78.2%), which was significantly higher than the PL-7 (+) (P < 0.001) and PL-12 (+) groups (P = 0.025). The median follow-up time was 10.7 months, during which no patients died. All received prednisone treatment, with or without immunosuppressants. At the 6-month follow-up, 96.3% of all patients (104/108) had a positive response to therapy, the JO-1 (+) and EJ (+) groups had a significantly higher improvement of forced vital capacity than the other 3 groups (P < 0.05), and the PL-7 group had the lowest FVC improvement (P < 0.05). The JO-1 (+) group and EJ (+) group had significantly higher anti-Ro-52 positive occurrence than the other 3 groups (P < 0.05).

**Conclusion:**

Anti PL-7 antibody had the same frequency as anti-JO-1 in ASSD-ILD, in which the ILD pattern was different with distinct anti-ARS antibodies. Most ASSD-ILD had a positive response to steroid therapies, with or without immunosuppressants. The PL-7 (+) group had the highest occurrence of UIP pattern, and a significantly lower response to therapy.

## Background

Anti-synthetase syndrome (ASSD) is a chronic autoimmune condition characterized by antibodies directed against an aminoacycl transfer RNA synthetase (ARS) along with a group of clinical features including the classical clinical triad: inflammatory myopathy, arthritis, and interstitial lung disease (ILD). Less commonly observed features include those defined as “accompanying findings”, which are Raynaud’s phenomenon, Mechanic’s hands, and fever. ASSD is heterogenous due to the different organs involved, the different onset timing of the triad, and the different response to steroids and immunosuppressive agents. There are no uniform criteria for the definition of ASSD;both Connor’s criteria [1] and Solomon’s criteria [2] are used in different cohorts, and the latter are based on the presence of myositis. However, as a major form of organ involvement in ASSD, ILD is responsible for the severity of ASSD, and is a main cause of function loss and morbidity in different studies, regardless of the ASSD [3, 4]. Myositis, as a member of the classic triad, might be absent at the time of ASSD diagnosis yet occur during follow up. If a triad finding appeared more than three months after the previous one, it was defined as an “ex-novo” finding [5].

In a recent published multi-centered study of 57 cases of ASSD-ILD, no differences in pulmonary functional progression were detected between patients positive for anti-JO-1 vs. non anti-JO-1 antibodies, while the subtype ILD forms were not discussed in this study [6]. We designed this retrospective cohort study to learn the disease behaviour of ASSD associated ILD, the subtypes of ASSD-ILD, and the response to medication of distinct ARS, using Connor’s criteria on ASSD published in *Chest* in 2010 [1].

## Methods

### Patients

We retrospectively recruited ILD patients with a final diagnosis of ASSD who were referred to the Department Respiratory and Critical Care Medicine of the Beijing Chaoyang Hospital from 2017.12 to 2019.3. All patients’ data were used anonymously. Informed consents was waived for this study due to its retrospective and observational nature. Patients with other connective tissue diseases including Rheumatoid Arthritis (RA), Systemic Sclerosis (SSc), Systemic Lupus Erythaematosus (SLE), and Sjögren Syndrome (SS), were excluded.

### Data acquisition

Data were obtained from the Electronic Medical Records (EMR). Patients recruited in this cohort had full data of blood tests, the whole autoimmune series, including the anti-ARS antibody series, chest High Resolution Computer Tomography (HRCT) scan, and Pulmonary Function Test (PFT) before treatment; and a biopsy via bronchoscopy (transbronchial lung biopsy or transbronchial cryobiopsy) if agreed upon by the MDT (Multidisciplinary Discussion Team), which was composed of 2 pulmonologists whose specialties were in ILD, 1 rheumatologist, 2 radiologists, 1 pathologist, 1 physician of occupational medicine, and held discussed every week. All the patients in this cohort had full data of at least one follow-up 6 months after diagnosis, when CT scan and PFT were applied to evaluate the effectiveness of treatment. Patients with a rapidly progressive ILD were excluded because the PFT could not be performed [7]. Patients age < 18 years old or > 80 years old, or with a history of malignancy were excluded. A positive response to therapy was defined as a minimal improvement of 5% FVC of predicted value, or an improvement of FVC of predicted value less than 5%, with an improvement of respiratory symptoms, including cough and dyspnoea, at the 6-month follow-up. Stability was defined as an improvement of FVC of predicted value less than 5%, without improvement of respiratory symptoms, and deterioration was defined as a decrease of FVC, or no FVC improvement with worsening of respiratory symptoms.

### Diagnostic criteria

Diagnosis of ASSD was based on criteria proposed by Connor et al. [1], which is a positive serologic testing for an anti-ARS autoantibody (JO-1, PL-7, PL-12, EJ, OJ) plus one or more of the following conditions: ① Evidence of myositis by Bohan and Peter criteria, ② Evidence of ILD, ③ Evidence of arthritis by clinical examination, radiographic findings, or patient self-report, ④ Unexplained, persistent fever, ⑤ Raynaud phenomenon, ⑥ Mechanic’s hand. The presence of ILD was evaluated by computed tomography (CT) and an MDT discussion. Radiological and pathological patterns of ILD were classified as usual interstitial pneumonia (UIP), nonspecific interstitial pneumonia (NSIP), or organizing pneumonia (OP) by the MDT routinely held every week. If the radiological features of the HRCT were typical and the MDT had a high confidence level (≥ 90%) on the radiological diagnosis, the MDT made a consensus of the ILD pattern without performing a biopsy on patients. RP-ILD (Rapidly Progressive ILD) was defined as a worsening of radiologic interstitial changes with progressive dysponea and hypoxaemia within 3 months after the onset of respiratory symptoms [7].

### Anti-ARS antibody analysis

The anti-ARS antibodies were identified using EUROIMMUN immunoblot according to the manufacturer’s instructions, including the anti-JO-1, anti-PL-7, anti-PL-12, anti-EJ, and anti-OJ antibodies. The results were considered positive if the bands showed a moderate or strong reaction.

### Statistical analysis

Statistical analysis was performed using SPSS software version 21.0. Chi-square and Fisher’s exact tests were used to compare the frequencies of the anti-ARS antibodies subgroups, ILD pattern, pulmonary function improvement, and anti-Ro-52 positivity rate.

## Results

From December 2017 to March 2019, 827 patients with ILD were screened in the study, 115 were positive for ARS antibodies, and 7 with rapidly progressed ILD were excluded (5 EJ positive and 2 PL-7 positive), as they were too weak to receive a PFT evaluation. Patients were divided into 5 groups according to the different ARS antibodies. All patients received treatment, with a base of prednisone of 0.5–1 mg/kg/d, with or without an immunosuppressive agents. Seventy-five patients (69.4%) received immunosuppressive agents, including cyclophosphamide (48 cases), Mycophenolate Mofetil (25 cases), and Tacrolimus (11 cases). Nine patients could not tolerate cyclophosphamide and were switched to Mycophenolate Mofetil (3 cases) and Tacrolimus (4 cases), whereas 2 had cyclophosphamide cessation and maintained monotherapy of prednisone. No patients died during the follow-up in this cohort with a median follow-up time of 10.7 months.

### Demographic features

Table 1 shows the general clinical characteristics of the 108 ILD cases with different positive anti-ARS antibodies. The median follow-up time was 10.7 months. The mean age was 56.8 ± 10.5 years. Two-thirds of patients were female (M:F = 36:72). No differences were observed between the demographic features of the 5 groups. No differences were detected of the baseline FVC and DLCO between groups (P = 0.582, P = 0.181, respectively).

**Table 1:** Demographic features

	Overall n = 108	JO-1 (+) n = 33	PL-7 (+) n = 30	EJ (+) n = 23	PL-12 (+) n = 13	OJ (+) n = 9	*P*
Age	56.8 ± 10.5	55.0 ± 11.4	58.3 ± 10.6	57.0 ± 9.1	57.5 ± 12.3	56.7 ± 8.5	0.794
Female	72 (66.1)	26 (78.8)	18 (60)	15 (65.2)	7 (53.8)	6 (66.7)	0.217
Mechanic’s hands	27 (25)	18 (54.5)	2 (6.7)	4 (17.4)	2 (15.4)	1 (11.1)	0.002
Clinical features							
Skin involvement (Gottron papules/heliotrope rash)	38 (35.0)	19 (57.6)	5 (16.7)	9 (39.1)	2 (15.4)	3 (33.3)	0.054
PM/DM	25 (23.1)	9 (27.3)	6 (20)	5 (21.7)	3 (23.1)	2 (22.2)	0.725
Anti-Ro-52	73 (67.6)	29 (87.9)	19 (63.3)	21 (91.3)	3 (23.1)	1 (11.1)	< 0.001
ILD pattern (radiological and pathological diagnosis by MDT)							
OP	53 (49.1)	18 (54.5)	8 (26.7)	18 (78.3)	4 (30.8)	5 (55.6)	
NSIP	42 (38.9)	13 (39.4)	15 (50)	4 (17.4)	8 (61.5)	2 (22.2)	
OP + NSIP	9 (8.3)	2 (6.1)	3 (10)	1 (4.3)	1 (7.7)	2 (22.2)	
UIP	4 (3.7)	0	4 (13.3)	0	0	0	
Positive Anti-Ro-52 antibody	73 (67.6)	29 (87.9)	19 (63.3)	21 (91.3)	3 (23.1)	1 (11.1)	
Baseline FVC (% predicted value)	67.1	71.2	68.5	63.3	70.2	64.9	0.582
Baseline DLCO (% predicted value)	68.0	65.3	59.7	62.6	57.6	63.6	0.181
Immunosuppressive agents use	75 (69.4)	22 (66.7)	20 (66.7)	18 (78.3)	9 (69.2)	6 (66.7)	N/A

### Frequency of distinct ARS

Of the 108 cases with ILD and positive ARS, 33 (30.6%) were anti-JO-1 positive, 30 (27.8%) were anti-PL-7 positive, 23 (21.3%) were anti-EJ positive, 13 (11.1%) were with anti-PL-12 positive, and 9 (8.3%) were anti-OJ positive (Table 1).

### Comparisons of clinical characteristics

Of the 108 cases of ASSD-ILD, mechanic’s hands were found in 27 (25%) patients, and the anti-JO-1 positive group had a significantly higher occurrence of mechanic’s hand (57.6%) than the other 4 groups (P = 0.002). Skin involvement (Gottron Papules and/or Heliotrope rash) was found in 38 (35.0%) patients, no differences were observed among the 5 groups (P = 0.054). Although not statistically significant, there was a clear trend with a higher frequency of DM-rashes in anti-JO-1 patients (57.6%) in comparison to that seen for anti-PL-7 (16.7%) and anti-PL-12 (15.4%). Polymyositis/Dermatomyositis (PM/DM) was diagnosed in 25 (23.1%) patients at the time of ASSD diagnosis, according to the Bohan and Peter criteria [8, 9], and no differences were observed in occurrence of the occurrence among the 5 groups (P = 0.725). Two patients were newly diagnosed with dermatomyositis 10 months after the diagnosis of ASSD, when they complained about fever and myalgia during follow up, and when the prednisone had been tapered down to 10 mg qd. Both of them were JO-1 positive, which could be considered as an “ex-novo” finding [5] (Table 1).

### Comparison of the ILD pattern

**Fig. 1:**
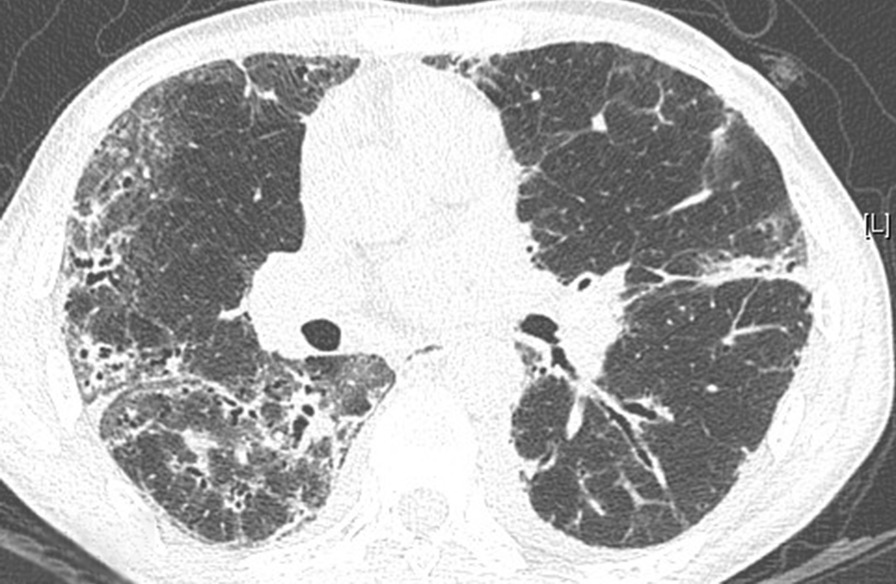
The CT of a male ILD patient in his 50s, who was anti-EJ positive, showing ground glass opacity, and traction bronchiectasis

**Fig. 2:**
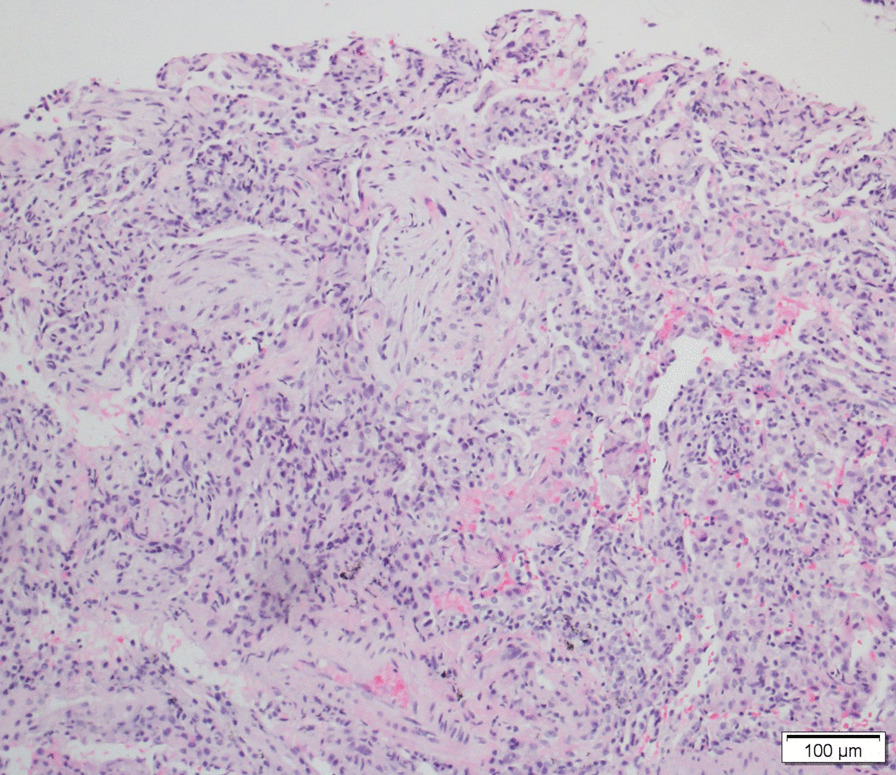
The right lower lobe cryobiopsy of this anti-EJ positive patient showed chronic interstitial inflammation and granulation tissue formation in alveoli (hematoxylin–eosin staining, original magnification × 100), in accordance with an organizing pneumonia (OP) pattern

**Fig. 3:**
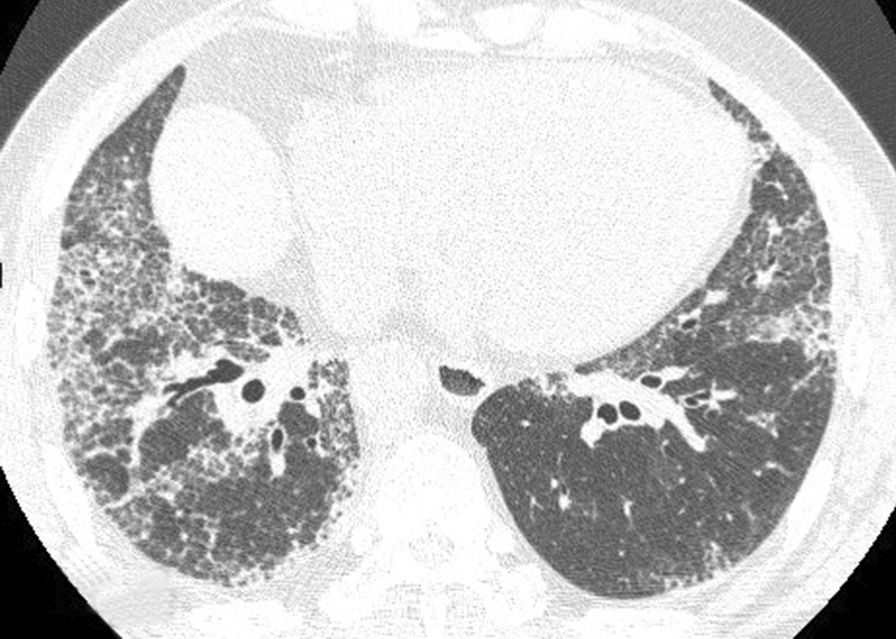
The CT of a male ILD patient in his mid 50s, who was anti-PL-7 positive, showing ground glass opacity, reticulation and traction bronchiectasis

**Fig. 4:**
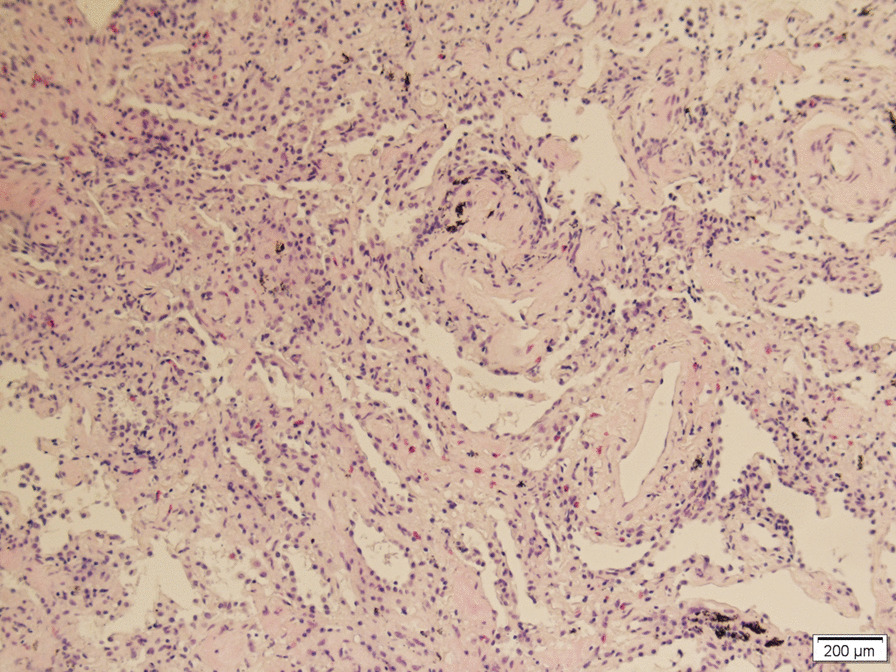
Right lower lobe cryobiopsy of this anti-PL-7 positive patient showed uniform chronic interstitial inflammation with lymphocyte infiltration and widened alveolar septum (hematoxylin–eosin staining, original magnification × 100), in accordance with a nonspecific interstitial pneumonia (NSIP) pattern

Of the 108 patients with ASSD-ILD, 30 cases received bronchoscopy for a transbronchial lung biopsy, 3 had bronchoscopy for a transbronchial cryobiopsy, to make a pathological diagnosis, and the radiological and pathological pattern of ILD was discussed by the MDT. The radiological pattern of the remaining 78 cases without biopsy was made by MDT with a high confidence level (> 90%). Of the 108 cases, 53 were an OP pattern (Figs. 1, 2), 42 were an NSIP pattern (Figs. 3, 4), 9 were an OP + NSIP pattern, and 4 were a UIP pattern. The 4 patients with the UIP pattern were all in the PL-7 group, which had a higher rate of UIP pattern than the other 4 groups, but no significant difference was observed. The anti-EJ positive group had the highest frequency of OP pattern (78.3%), which was significantly higher than the PL-7 (P < 0.001) and PL-12 groups (P = 0.025). The JO-1 group had a higher occurrence of OP pattern than the PL-7 group (P = 0.019) (Tables 1, 2).

**Table 2:** Comparison of the OP pattern

	EJ (+) n = 23	JO-1 (+) n = 33	PL-7 (+) n = 30	PL-12 (+) n = 13	OJ (+) n = 9
JO-1 (+)	0.068	–	–	–	–
PL-7 (+)	< 0.001	0.019	–	–	–
PL-12 (+)	0.025	0.208	0.909	–	–
OJ (+)	0.397	1.000	0.203	0.567	–

### Comparison of the PFT improvement

All patients were treated with prednisone of 0.5–1 mg/kg/day as the initial dose, with or without immunosuppressive agents. Each patient had at least one follow-up 6 months after diagnosis. The PFT (FVC, DLCO) improvement of the 6-month follow-up was recorded (Table 3).

**Table 3:** Response to Treatment and Improvement of PFT

Improvement of PFT (mean ± Std)	JO-1 (+) n = 33	PL-7 (+) n = 30	EJ (+) n = 23	PL-12 (+) n = 13	OJ (+) n = 9
Change of FVC% predicted value (%)	8.6 ± 4.4	1.1 ± 0.7	5.5 ± 3.8	2.3 ± 1.8	2.7 ± 1.6
Chang of DLCO% predicted value (%)	16.9 ± 9.8	9.1 ± 4.5	10.5 ± 5.4	10.0 ± 4.9	10.3 ± 4.8
Positive response to therapy	33 (100)	27 (90)	23 (100)	12 (92.3)	9 (100)
Stable with therapy	0	3 (10)	0	1 (7.7)	0
Deterioration	0	0	0	0	0

In the follow-up, no patients experienced deterioration. All patients in the JO-1 (+), EJ (+), and OJ (+) groups, 90% in the PL-7 (+) group and 92.3% in the PL-12 (+) group had positive responses (104/108, 96.3%) to therapy. Three (10%) patients in the PL-7 (+) group and 1 (7.7%) in the PL-12 (+) group showed stability with therapy. The improvement of FVC: at 6 months of follow up, the anti-JO-1 postive group had a significantly higher FVC improvement than the anti-PL-7 positive group (P < 0.001), anti-PL-12 positive group (P < 0.001), and anti-OJ positive group (P = 0.004); the anti-EJ positive group had a significantly higher FVC improvement than the anti-PL-7 positive group (P < 0.001), anti-PL-12 positive group (P = 0.001), and anti-OJ positive group (P = 0.005). The anti-PL-7 positive group had the lowest FVC improvement among all groups, and it was significantly lower than the anti-PL-12 positive group (P = 0.008), anti-OJ positive group (P = 0.006), anti-EJ positive group and anti-JO-1 positive group as described previously. The FVC improvement between the anti-OJ-positive and anti-PL-12 positive groups was not significant (P = 0.527). The FVC improvement between the anti-JO-1 positive and anti-EJ positive groups was not significant (P = 0.966) (Table 4).

**Table 4:** Comparison of the FVC improvement

Improvement of FVC% predicted value (%)	EJ (+) (5.5 ± 3.8)	JO-1 (+) (8.6 ± 4.4)	PL-7 (+) (1.1 ± 0.7)	PL-12 (+) (2.3 ± 1.8)	OJ (+) (2.7 ± 1.6)
JO-1 (+)	0.966	–	–	–	–
PL-7 (+)	< 0.001	< 0.001	–	–	–
PL-12 (+)	0.001	< 0.001	0.008	–	–
OJ (+)	0.005	0.004	0.006	0.527	–

The improvement of DLCO: The anti-JO-1 positive group had a significantly higher improvement of DLCO than the anti-PL-7 positive group (P = 0.011), anti-EJ positive group (P = 0.008), and anti-PL-12 positive group (P = 0.023). The DLCO improvements of the other between-group differences were not significant (P > 0.05) (Table 5).

**Table 5:** Comparison of the DLCO improvement

Improvement of DLCO % predicted value (%)	EJ (+) (10.5 ± 5.4)	JO-1 (+) (16.9 ± 9.8)	PL-7 (+) (9.1 ± 4.5)	PL-12 (+) (10.0 ± 4.9)	OJ (+) (10.3 ± 4.8)
JO-1 (+)	0.008	–	–	–	–
PL-7 (+)	0.432	0.011	–	–	–
PL-12 (+)	0.783	0.023	0.312	–	–
OJ (+)	0.907	0.063	0.455	0.903	–

### Comparison of the myositis-associated antibodies

Anti-Ro-52 antibodies were found in 73 (67.6%) cases, and the anti-JO-1 positive group and anti-EJ positive group had significantly higher anti-Ro-52 positive rates than the other 3 groups (P < 0.05) (Table 6).

**Table 6:** Comparison of the Anti-Ro-52 antibody

Positive Anti-Ro52 antibody	EJ (+) (91.3%)	JO-1 (+) (87.9%)	PL-7 (+) (63.3%)	PL-12 (+) (23.1%)	OJ (+) (11.1%)
JO-1 (+)	1.000	–	–	–	–
PL-7 (+)	0.019	0.022	–	–	–
PL-12 (+)	< 0.001	< 0.001	0.015	–	–
OJ (+)	< 0.001	< 0.001	0.018	0.878	–

## Discussion

In this study we compared the clinical features and disease behaviors of ASSD-ILD patients with different ARS. The JO-1 group had a significant higher rate of mechanic’s hand (57.6%) than the other 4 groups. PM/DM was diagnosed in 25 (23.1%) patients and no difference were observed among the 5 groups. The anti-PL-7 positive group had a higher frequency of UIP pattern (13.3%) than the other 4 groups, but the difference was not significant, and the EJ group had the most frequent OP pattern (78.2%), which was significantly higher than the PL-7 (P < 0.001) and PL-12 groups (P = 0.025). All received prednisone treatment, with or without immunosuppressants, at the 6-month-follow up, the JO-1 and EJ groups had a significantly higher improvement of forced vital capacity than the other 3 groups (P < 0.05), and the PL-7 group had the lowest FVC improvement (P < 0.05).

ASSD was first defined by Marguerie in 1990 [10] as a relatively homogeneous syndrome comprised of inflammatory myopathy, pulmonary fibrosis, and arthritisin a retrospective cohort of 29 patients, including 19 anti-JO-1 positive, 4 anti-PL7 positive, and 6 anti-PL-12 positive cases. JO-1 is the most frequent antibody in all the ASSD studies and represents the most common form [11]. In our cohort,PL-7 (30/108) had a similar frequency as JO-1 (33/108). Studies have shown that specific ARS have different ASSD phenotypes, such as skin involvement (heliotrope rash) being more frequent in anti-PL7 positive patients [3, 12]. However, few studies have been performed to understand the correlation between different ARS antibodies and the ILD pattern, which is a common manifestation of ASSD and a main cause of death.

Overall, 35–45% of patients diagnosed with PM/DM will be afflicted with ILD during the course of myositis [1], and some have reported an ILD prevalence of 65% in PM/DM [13]. ILD occurs after the diagnosis of PM/DM in up to 40% of patients, and precedes the diagnosis of PM/DM in 20–30% of cases [14, 15], causing morbidity and mortality. OP, NSIP, and mixed NSIP-OP patterns are more frequent than UIP patterns in PM/DM-ILD [13]. ARS antibodies, one of the myositis specific antibodies (MSA), are positive in 30–45% of patients with a myopathic inflammatory disease [3, 16], and PM/DM patients with positive ARS have higher prevalence of ILD than those without such antibodies. As on 2015, ARS can also be positive in ILD patients who do not meet the criteria of inflammatory myositis of any other CTDs, who are considered as idiopathic interstitial pneumonitis (IIP) with positive ARS antibodies, or can be classified as interstitial pneumonia with autoimmune features (IPAF) [17]. ASSD is a term that focuses more on the ARS antibody; however, to date, there are no uniform diagnostic criteria of ASSD. In some cohorts the ASSD was defined by the presence of myositis [3], while Connor’s ASSD criteria proposed in 2010 uses a positive ARS antibody plus one or more of myositis, ILD, arthritis, fever, Raynaud, or Mechanic’s hands, which is more pragmatic [1]. In patients with ASSD, the classic clinical triad (myositis, ILD, arthritis) might have different onset times. In the large cohort of 828 ASSD patients (AENEAS collaborative group) [5], the onset mainly began with a single triad findings, and some cases presented as one/two triad findings in the clinical time course [5], similar to our study.

ARS is a group of antibodies targeting the ribonucleoproteins involved in protein synthesis, and eight anti-ARS Abs have been described: anti-histidyl (anti-JO-1), anti-threonyl (anti-PL-7), anti-alanyl (anti-PL-12), antiglycyl (anti-EJ), anti-isoleucyl (anti-OJ), anti-asparaginyl (anti-KS), anti-phenylalanyl (anti-Zo), and anti-tyrosyl (anti-Ha) tRNAs, the former 5 of which are tested routinely in clinical practice.

Studies have been applied to explore the significance of distinct ARS antibodies and the results have been different. JO-1 is the most prevalent antibody in either ASSD (60–75%) [18, 19] or inflammatory myositis (found in 20–30% of PM patients and in 5–10% DM patients) [2, 18]. A cross-sectional and longitudinal analysis in 77 patients with inflammatory myositis associated ILD showed that the anti-JO-1 positive patients [28] had worse lung function and CT scores over time compared to those without detectable ARS antibodies [20]. Meanwhile, another retrospective study of 202 cases of ASSD showed that the 5- and 10-year unadjusted cumulative survivals were 90% and 70% for anti-JO-1 positive patients, respectively, which were significantly better than that of non-JO-1 patients (P < 0.005) [19]. However, in this cohort, the most common cause of death was pulmonary fibrosis (49%), which was similar between JO-1 and non-JO-1 patients (P = 0.511) [19]. In another cohort of 43 patients with ASSD associated ILD, 6 (14%) patients had died at 5 years, and the anti-JO-1 positive rate was significantly higher in survivors (86%) than that of the deceased patients (50%),who had a significantly lower baseline FVC [21]. In our cohort, the baseline FVC and DLCO of the distinct ARS antibody groups had no differences while the improvement with therapy had differences.

Anti-PL-7/PL-12 positivity in ASSD patients was found associated with more aggressive ILD and decreased survival as compared with those with anti-JO-1 antibodies. An ASSD cohort included 75 JO-1 (+) cases, 15 PL-7 (+) cases, and 5 PL-12 (+) cases, and the anti-PL-7/PL-12 positive patients had more ILD compared with those with anti-JO-1 antibodies (90% vs. 68%). Anti-JO-1 antibody results in more severe myositis, joint impairment and increased risk of cancer [22]. In a cohort of 7 cases of anti-PL-7 positive ASSD-ILD, lung biopsy revealed 50% of cases with UIP [23].In another retrospective study of 20 cases of anti-JO-1 positive ASSD-ILD, 35% of cases had a UIP pattern on lung biopsy [24]. In a cohort of 12 anti-PL-7 positive ASSD patients, the mean age at the first sign of clinical symptoms was 56.3 years, which was similar with our study, all presented with ILD, in which 9 had an NSIP pattern, 2 had an OP pattern and 1 had an obliterative bronchiolitis (BO) pattern [25]. In another anti-PL-7 positive ASSD cohort with 18 patients, all had myositis when first diagnosed, and 10 (55.6%) had ILD [26].

In an anti-PL-12 positive ASSD cohort with 17 patients, the mean age at diagnosis was 60.3 years, all patients had ILD when diagnosed, 15 of which had the NSIP pattern and 2 the OP pattern, and 7 had mild myositis [27].

Another anti-PL-12 positive ASSD cohort with 31 patients had a myositis prevalence of 52% (16/31), and an ILD prevalence of 90% (28/31), 14 out of which had a UIP pattern, 5 an NSIP pattern and 5 an OP pattern; this was confirmed histopathologically in 14 patients who either received surgical lung biopsy, or evaluated by HRCT [28].

In contrast, studies with larger samples suggest that ASSD with various ARS is relatively homogenous; however, the distribution and timing of myositis, ILD alone at onset, and rashes differ among patients. In a retrospective Japanese cohort of ASSD with 166 patients, ILD alone at onset was 63% in the OJ (+) group, 33% in the PL-12 (+) group, 26% in the EJ (+) group, 14% in the PL-7 (+) group, and 5% in the JO-1 (+) group [12], and those with anti-JO-1, anti-EJ, and anti-PL-7 developed myositis later if they had ILD alone at the time of disease onset. In the AENEAS cohort with 828 ASSD patients, characteristics of the triad findings were similar and the onset mainly began with a single triad finding in all groups despite some differences in overall prevalence. The PL-7 (+) group and EJ (+) group had higher ILD prevalence compared with the JO-1 (+) group (P = 0.001, P = 0.005, respectively), and the EJ (+) group presented more frequent acute onset (74%), which was defined as dysponea progressing rapidly in 4 weeks from respiratory symptom onset. Moreover, survival was not influenced by the distinct anti-ARS antibody’ positivity, suggesting that ASSD is a heterogeneous condition and antibody specificity only partially correlates with the clinical course [5].

However, the pulmonary function of ASSD-ILD responding to therapy, one of the disease behaviours, had never been studied previously. The change of FVC, one of the most important factors of the ILD clinical course, had been used to classify ILD into a reversible or progressive type in classification and clinical trials [29, 30]. Most of the patients (96.3%) had a positive response to therapy and an improvement in FVC in our study. The remaining (3.7%) patients were stable after therapy, which means the ARS antibody is a treatable trait of steroids or immunosuppressants for ILD, even without the presence of myositis. The myositis prevalence showed no differences among the 5 groups, which made the FVC comparable between groups, for respiratory muscle weakness impacts the spirometry values, leading to a complicated interpretation [1]. No standard treatment has been proposed for ASSD, however, prednisone should be the mainstay, with or without immunosuppressants [31], which was proved again by our study.

Anti-Ro-52, a myositis associated antibody, showed a positive frequency of 65% in an anti-JO-1 positive ASSD cohort [32]. In our cohort, the anti-Ro-52 positivity rate was 87.9% in the JO-1 (+) group, and 91.3% in the EJ (+) group, which was significantly higher than the other 3 groups. The EJ (+) and JO-1 (+) groups had the highest frequency of OP pattern, indicating that the occurrence of OP might be correlated with anti-Ro-52 positivity.

Our study had several limitations. It was single centre and retrospective, the sample was relatively small and the follow up time was not sufficiently long. In another ASSD cohort, the RP-ILD was statistically more prevalent in patients with positive anti-PL-7 antibodies than those without anti-PL-7 [3]. The RP-ILD cases were not included in this study because spirometry could not be performed upon diagnosis due to the patients’ weakness; in our study, the excluded 7 cases of RP-ILD included 5 with EJ positivity and 2 with PL-7 positivity. Shi’s cohort, using Solomon’s criteria of ASSD, showed that a coincidence of anti-Ro-52 antibody predicted RP-ILD [3]. RP-ILD is more life threatening and requires more study in the future.

## Data Availability

The datasets generated and analyzed during the current study are not publicly available because the patients are still in close follow up to collect further data, but are available from the corresponding author on reasonable request.

## Abbreviations

ASSD: Anti-Synthetase Syndrome
ARS: Aminoacyl tRNA synthetases
HRCT: High Resolution Computer Tomography
FVC: Forced vital capacity
ILD: Interstitial lung disease
NSIP: Non-specific Interstitial Pneumonitis
OP: Organizing Pneumonia
UIP: Usual Interstitial Pneumonia
MDT: Multidisciplinary discussion team
PM: Polymyositis
DM: Dermatomyositis
